# Pan-cancer analysis of Methyltransferase-like 16 (METTL16) and validated in colorectal cancer

**DOI:** 10.18632/aging.206210

**Published:** 2025-02-27

**Authors:** Ling Liu, Siying Wang, Xuyu Chen, Qian Luo, Zhaoxia Wang, Juan Li

**Affiliations:** 1 Department of Oncology, The Second Affiliated Hospital of Nanjing Medical University, Nanjing, Jiangsu 210011, China

**Keywords:** METTL16, pan-cancer, biomarker, colorectal cancer, tumorigenesis

## Abstract

Human Methyltransferase-like 16(METTL16) is an independent N6-methyladenosine (m6A) methyltransferase. Previous studies have proven METTL16 been linked with some types of cancers. However, comparative studies of the relevance of METTL16 across diverse tumors remain sparse. We comprehensively investigated the effect of METTL16 expression on tumor prognosis across human malignancies by analyzing multiple cancer-related databases like Tumor Immune Estimation Resource (TIMER) and human protein atlas (HPA). Bioinformatics data indicated that METTL16 was overexpressed in most of these human malignancies and was significantly associated with the prognosis of patients with cancer, especially in colorectal cancer (CRC). Subsequently, *In vitro* experiments, the utility of METTL16 that downregulation of its expression could result in reduced proliferation and migration of CRC cells. Our findings reveal novel insights into METTL16 expression and its biological functions in diverse cancer types, indicating that METTL16 could serve as a prognostic biomarker and plays an important role in colorectal cancer.

## INTRODUCTION

Gastric cancer (GC) is a significant global health concern, annually affecting about one million individuals and resulting in approximately 769,000 deaths [1]. Genomic and epigenomic researches are crucial to better proper understanding and recognition of process of tumorigenesis. A pan-cancer analysis of novel genes will be helpful for playing a constructive role in cancer development.

METTL16 (Methyltransferase-like 16) protein, also known as: METT10D, Gene ID: 79066, has recently been reported as an independent RNA methyltransferase [2]. N6-methyladenosine (m6A) is the most popular mRNA modification in mammals and has been involved in various biological processes. METTL16 is involved in this m6A Methylation regulation [3]. METTL16 controls abundant m6A installers by regulating S-adenosylmethionine (SAM) homeostasis to affect multiple aspects of human. The appearance of the m6A methylation alters multiple biological processes and may result in cancer development and progression when it interacts tumor-related genes [4]. Similarly, METTL16 has been associated with many functions such as proliferation and differentiation in tumors. The METTL16 regulators have been connected with various types of cancers [5–8]. However, current studies only explored that METTL16 was linked to a few cancer types, and its biological role remains unexplored in most tumor types.

To explore the expression state of METTL16 among various cancer types in a pan-cancer analysis, we concluded survival status, the subcellular distribution, genetic alteration and relevant molecular pathways. In the results, we found that METTL16 was highly expressed in colorectal cancer (CRC)and was associated with the prognosis of the disease, so we chose CRC as the cancer to further explore. *In vitro* experiments, the downregulation of METTL16 could result in reduced proliferation and migration of CRC cells. Our study fills a critical knowledge gap by revealing the biological function of METTL16 and highlighting its potential as a prognostic biomarker for CRC.

## MATERIALS AND METHODS

### METTL16 expression analysis

We used TIMER2 (tumor immune estimation resource, version 2) (http://timer.cistrome.org/) database [9] to compare the expression of METTL16 between tumor and adjacent normal tissues among 33 types of cancers. HPA dataset (https://www.proteinatlas.org/) was used to conclude the RNA expression across different organs and cell lines compared METTL16 expression in normal tissues and tumors tissues.

### Survival prognosis analysis

The PrognoScan database (http://dna00.bio.kyutech.ac.jp/PrognoScan/index.html) is an online database tool containing differential expression gene data and clinical data to assess the prognostic value of specific genes. Patient cases were divided into high expression and low expression depend on the gene expression level. We obtained the overall survival data to investigate the association between METTL16 expression and survival status by Kaplan–Meier Plotter across various tumors.

### Immunohistochemistry (IHC) staining

We get Immunohistochemistry staining images of METTL16 from HPA database. METTL16 was stained by antibody HPA020352. Staining intensity (negative, weak, moderate, or strong) and the fraction of stained cells (<25%, 25–75%, or >75%) are provided in the HPA website to evaluate differences in METTL16 expression at the protein level.

### Subcellular distribution

HPA dataset was used to conclude the subcellular distribution of METTL16 in different cell lines. Immunofluorescent staining of human cell shows localization to nucleoplasm and cytosol.

### Immune infiltration analysis

First, we verified the correlation between expression of MRTTL16 and six immune cells via TIMER2 database. TIMER2 is a comprehensive database for systematical analysis of immune infiltrates across diverse cancer types. TIMER2 provides immune infiltrates' abundances estimated from the TCGA database by multiple immune deconvolution methods to explore tumor immunological features comprehensively. The infiltration data were used to demonstrate if there was a link between METTL16 expression and infiltration. TISIDB online platform [10] was used to analyze the association between TILs (tumor-infiltrating lymphocytes) abundance and METTL16 expression.

### Genetic alteration analysis

cBioPortal tool (https://www.cbioportal.org/) was used to analyze the condition of alteration frequency, mutated site information and mutation type across all TCGA tumors. With or without METTL16 genetic alteration, overall survival (OS) and disease-free survival (DFS) were compared across all the TCGA cancer types. GSCA (Gene Set Cancer Analysis) (https://guolab.wchscu.cn/GSCA/#/) was used to evaluate the impact of the DNA copy number amplification on METTL16 expression.

### Gene enrichment analysis

We used STRING tool [11, 12] for the relevant analysis of protein-protein interaction network. The main settings were: meaning of network edges(“evidence”), minimum required interaction score: (“medium confidence (0.400)”), max number of interactors to show (“no more than 10 interactors” in 1st shell), active interaction sources: (“text mining, experiments, databases, co-expression, neighborhood, gene Fusion and co-occurrence”). We concluded string tool data to perform GO and KEGG pathway analysis.

### CeRNA network construction

We used starBase3.0 [13], TargetScan3.0 [14] and miRDB to predict the target miRNA of METTL16. Subsequently, we analyzed the correlation between METTL16 expression and target miRNA expression to confirm the miRNAs that are consistent with ceRNA conditions. The miRNAs include hsa-miR-340-5p, hsa-miR-506-3p and hsa-miR-1252-5p. Finally, we used starBase3.0 website to predict the potential binding site between METTL16 and target miRNAs. We used starBase and miRNet2.0 [15, 16] to predict the target lncRNA. Subsequently, we analyzed the correlation between the three-target miRNA expression and lncRNAs expression to confirm the lncRNAs that are consistent with ceRNA conditions. The positive correlation between mRNA and lncRNA expression levels is crucial to establish lncRNA-miRNA-mRNA (METTL16) ceRNA network. Furthermore, we explored the subcellular localization of the lncRNAs by finding data in the lncLocator [17, 18].

### Cell culture and transfection

The COAD cell lines including SW480, SW620 and the normal cell line (NCM460) were all obtained from The Second Affiliated Hospital of Nanjing medical university laboratory. All cell lines were authenticated by STR profiling and were routinely tested for mycoplasma contamination. All cell lines were cultured in EMEM supplemented with 10% FBS. The knockdown of METTL16 was achieved by transfecting cells with siRNAs using Lipofectamine 3000 (Invitrogen, Carlsbad, CA, USA). METTL16 siRNA (PROTEINBIO, Nanjing, China) was transfected in the SW480 and SW620 cell lines. All experimental followed the manufacturer’s instructions.

### Quantitative real-time PCR

We used TRIzol reagent (Invitrogen, USA) to extract the total RNA and the HiScript III All-in-One RT SuperMix Perfect for qPCR (Vazyme, Nanjing, China) to perform reverse transcription. The total RNA was extracted using TRIzol reagent (Invitrogen, USA), and reverse transcription was performed using the HiScript III All-in-One RT SuperMix Perfect for qPCR (Vazyme, China) to obtain complementary DNA (cDNA). The expression levels of METTL16 and GAPDH were measured by quantitative real-time PCR (RT-qPCR) utilizing the ChamQ Universal SYBR qPCR Master Mix (Vazyme, Nanjing, China). The primers were listed below: si-METTL16#1:5′-AUGGCUGGUAUUUCCUCGCAATT-3′; si-METTL16#2:5′-GGAAGAUUUUGGACUUUCUTT-3′.

**Table:** 

**Primer sequences**	
**Gene**	**Sequence (5′→3′)**
*METTL16*	F: TGAGAGGTGGCTGTTGGTC
	R: AGTGAGCTAAGATCGCACCA
*GAPDH*	F: GAAGGTGAAGGTCGGAGTC
	R: GAAGATGGTGATGGGATTTC

### Cell proliferation assays

SW480 and SW620 cell lines (2000 cells/well) cells were seeded in 96-well plates with siRNAs. CCK-8 reagent (Vazyme, Nanjing, China) was added into six well plate and incubated at 37°C for 2 h. We used a microplate reader measuring the absorbance at 450 nm. SW480 and SW620 cells were planted into six well plates exposed to siRNA to perform colony formation assays. After a 2-week incubation period, the cells were fixed with 4% paraformaldehyde and staining with crystal violet.

### Transwell assay

CRC cells were seeded in the upper chambers of transwell plates and the lower chamber was subsequently filled with DMEM supplemented with 15% FBS. Following incubation at 24 hours, the cells on the lower surface were fixed with 4% paraformaldehyde and then stained with crystal violet. Finally, we used a microscope to enumerate the cells.

### Statistical analysis

All experiments were conducted using GraphPad Prism 9, and the results were displayed as means ± SD derived from three separate samples. To assess differences, an unpaired two-tailed *t*-test, as recommended for independent analysis, was employed. *P* < 0.05 was considered statistically significant.

## RESULTS

### Expression levels METTL16

**Figure 1:**
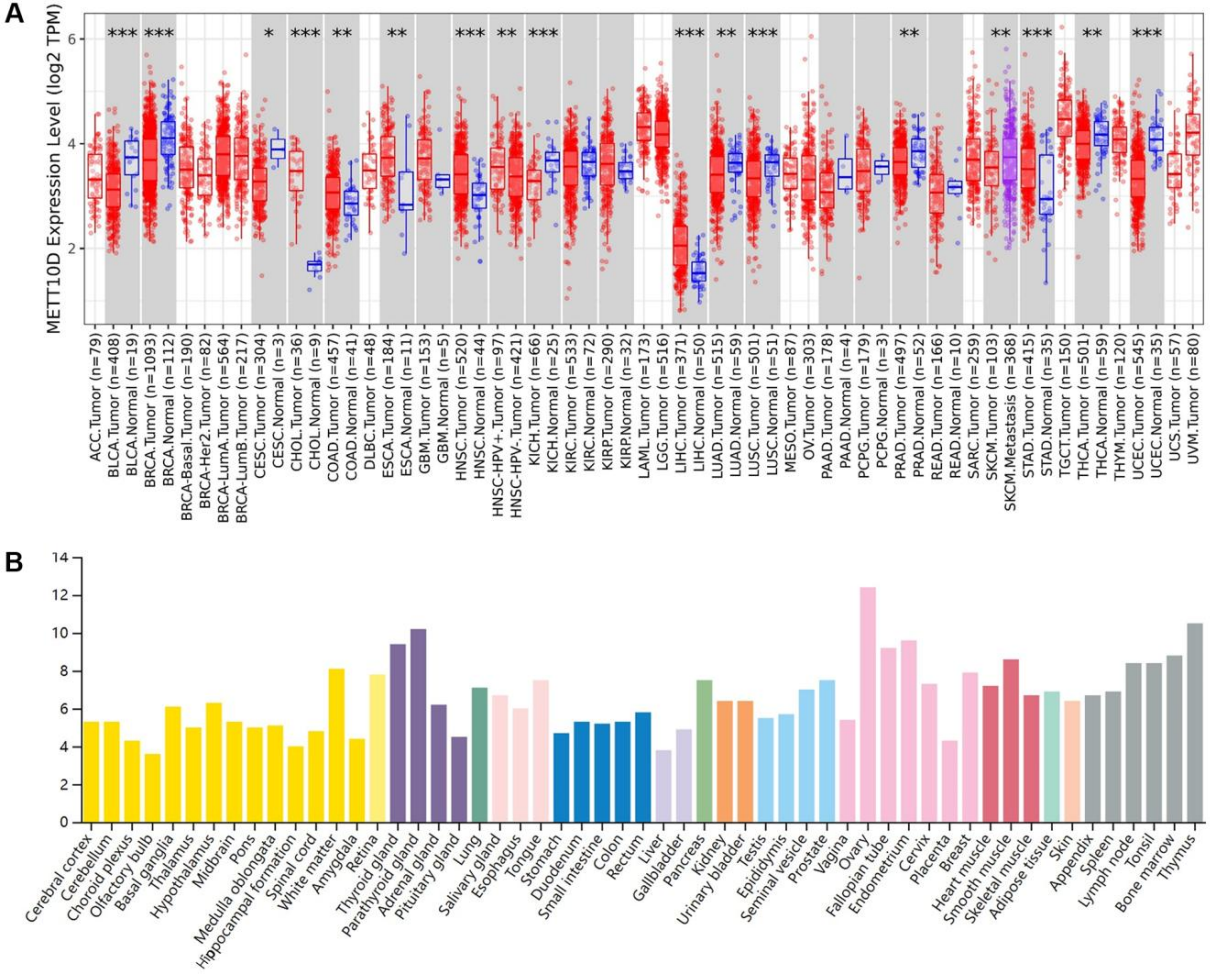
**Expression level of METTL16 in human tumors.** (**A**) Expression level of METTL16 in TCGA tumors vs. adjacent tissues as visualized by TIMER2. ^*^P < 0.05; ^**^P < 0.01; ^***^P < 0.001. (**B**) The RNA expression level across different organs in HPA database.

As an independent N6-methyladenosine (m6A) methyltransferase, we provided a comprehensive analysis regarding the crucial role of human METTL16 (Supplementary Figure 1). We used the TIMER2 “Gene DE” module to explore the differential expression of METTL16 across all TCGA tumors compared with corresponding adjacent normal tissues (if available). As shown in Figure 1A, the expression level of METTL16 in the tumor tissues of cholangiocarcinoma (CHOL), head and neck squamous cell carcinoma (HNSC), liver hepatocellular carcinoma (LIHC), Stomach adenocarcinoma (STAD) (*P* < 0.001), colon adenocarcinoma (COAD), esophageal carcinoma (ESCA) (*P* < 0.01), is higher than the corresponding control tissues. Meanwhile, METTL16 showed lower expression in bladder urothelial carcinoma (BLCA), breast invasive carcinoma (BRCA), kidney chromophobe (KICH), lung squamous cell carcinoma (LUSC), uterine corpus endometrial carcinoma (UCEC) (*P* < 0.001), lung adenocarcinoma (LUAD), prostate adenocarcinoma (PRAD), thyroid carcinoma (THCA) (*P* < 0.01) and cervical squamous cell carcinoma and endocervical adenocarcinoma (CESC) (*P* < 0.05) compared with the corresponding control tissues. HPA dataset was used to conclude the RNA expression across different organs and cell lines compared METTL16 expression in normal tissues and tumors tissues (Figure 1B and Supplementary Figure 2).

### Analysis of link between METTL16 expression level and prognosis

**Figure 2:**
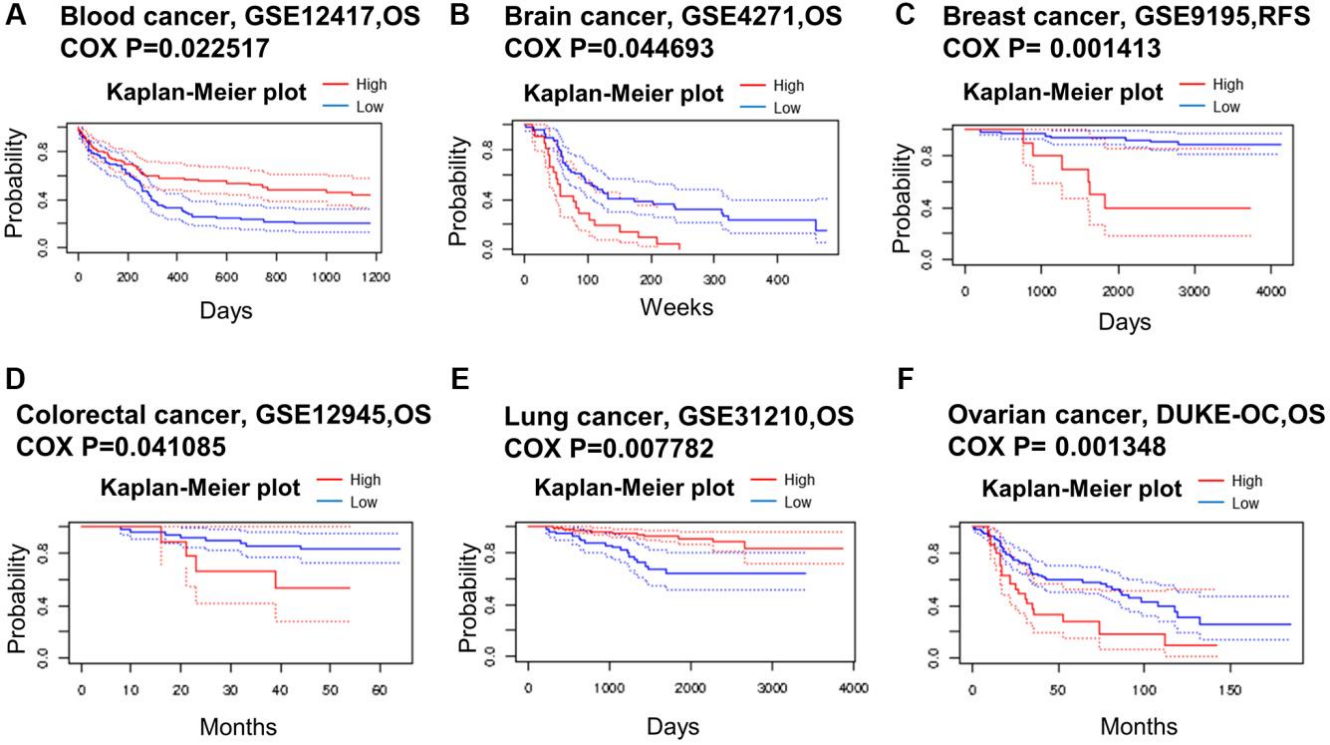
**Kaplan–Meier survival curves comparing high and low expression in multiple cancer types from PrognoScan.** (**A**) Blood cancer. (**B**) Brain cancer. (**C**) Breast cancer. (**D**) Colorectal cancer. (**E**) Lung cancer. (**F**) Ovarian cancer. A *p*-value < 0.05 was considered a significant threshold.

We used data from the PrognoScan database to assess the relationship between METTL16 expression levels and prognosis in multiple cancer types. We divided cancer samples into high expression group and low expression group according to the expression level of METTL16 (Figure 2). The high expression of METTL16 in brain cancer, colorectal cancer and ovarian cancer was associated with shorter OS, suggesting that METTL16 plays a carcinogenic role in these tumors. Conversely, in blood cancer and lung cancer, overexpression of METTL16 was associated with longer OS, suggesting that METTL16 plays a protective role. These findings reflect the diversity of roles of METTL16 in tumorigenesis and progression.

Interestingly, there were apparent connection of METTL16 expression between age, gender, stage as well as race (Supplementary Figure 3). Next, we evaluated the differential expression of METTL16 in patients with different tumor types in terms of clinical information by TIMER2 tools. The results showed that METTL16 is associated with clinical features (ages, race, stage and gender) in some cancer such as BRCA, PAAD and UVM. Therefore, these results demonstrated that METTL16 may be a potential prognostic marker in various cancers.

### Subcellular distribution date

We used HPA dataset to analyze the subcellular distribution of METTL16 in different cell lines such as A431-16, U-2-OS and U-251MG. The METTL16 mainly localized to the nucleoplasm and additional localized to the cytoplasm (Supplementary Figure 4). Staining patterns of the cytosol vary from smooth to granular, and the staining is stronger close to the nucleus.

### Correlation analysis between METTL16 expression and immune cells

Immune cells play important roles in regulating tumor development [19, 20]. M6a RNA methylation is an emerging epigenetic modification, and its potential role in immunity remains unknown. We assessed the connection between METTL16 expression and the degree of immune infiltration across various cancer types. We found that it exists a strong genetic correlation between METTL16 expression levels and the expression degree of immune cells (B cell, CD4+ T cell, CD8+ T cell, neutrophil, macrophage and myeloid dendritic cell) (Supplementary Figure 5). Therefore, the results indicated that expression of METTL16 was correlated with immune infiltration in cancers, which may lead to the development of related tumor diseases.

### Genetic and epigenetic alteration analysis

**Figure 3:**
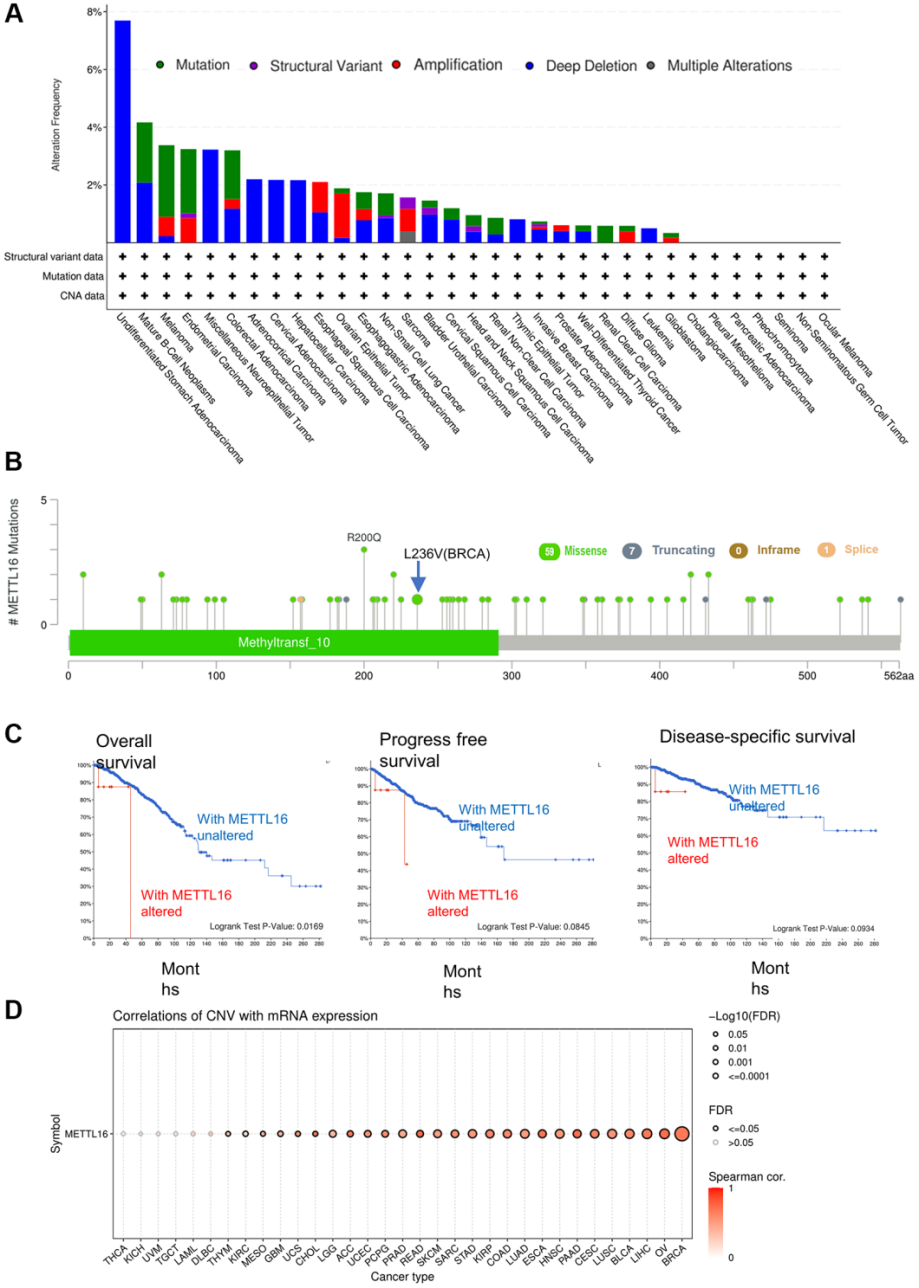
**Mutation status of METTL16 across TCGA tumors.** (**A**) The alteration frequency with mutation type. (**B**) Mutation site (**C**) The association between mutation and OS (Overall survival), PFS (Progression-free survival) and DSS (Disease-specific survival). (**D**) A Spearman association between METTL16 CNV and mRNA was performed in pan-cancer.

Growing evidence shows that altered patterns of gene expression are the significant cause of cancers [21]. Next, we explored the METTL16 genetic alterations in human tumor samples via the cBioPortal tools. Data reflect the fact that overall gene mutation frequency of METTL16 in these tumors was relatively medium. Melanoma had the highest mutation alteration frequency of METTL16 and the ovarian epithelial tumor had the highest frequency of METTL16 alteration of amplification (Figure 3A). Subsequently, we displayed overall mutations and their location of METTL16 (Figure 3B). To test whether a link between genetic alterations of METTL16 and the clinical prognosis of patients, we divided cancer samples into altered group and unaltered group. We found that with genetic alteration of METTL16 showed a poor prognosis in overall survival (*P* = 0.0169), progress free survival (*P* = 0.0845) and disease-specific survival (*P* = 0.0934) (Figure 3C). Next, we used GSCA website to explore the potential connection between CNV (copy number variations) and METTL16 expression across the 33 types of cancers. It found that METTL16 was positively correlated with CNV across 27 types of cancers (Figure 3D).

### Proliferation and metastasis biomarkers analysis

**Figure 4:**
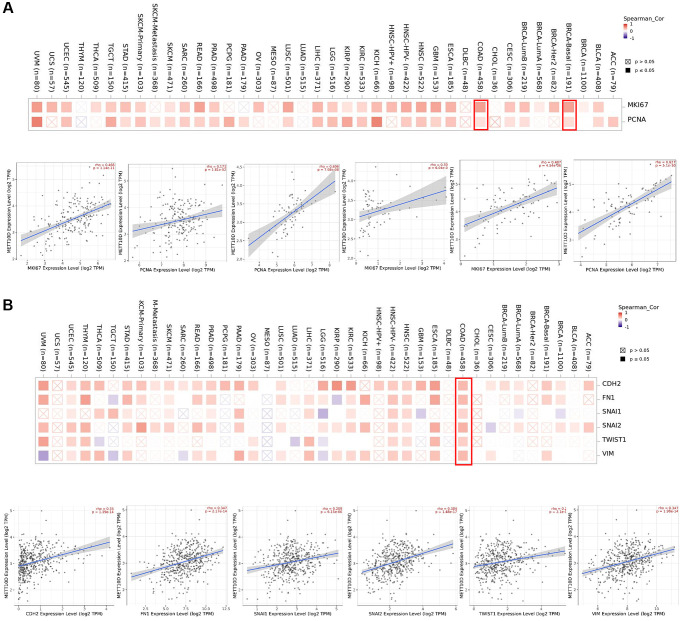
**Correction analysis between METTL16 and cell proliferation and EMT.** (**A**) Associations between METTL16 expression level and proliferation markers (MKI67 and PCNA) were investigated in different cancer types. (**B**) Correlation analysis on the association betweenMETTL16 expression and EMT markers (CDH2, Fibronectin 1, Snail1, Snail2, TWIST1and Vimentin).

Various biological processes such as proliferation and metastasis could play key roles throughout the course of tumor development. We analyzed the correlation between METTL16 and the classic proliferation markers, MKI67 and PCNA [22]. ln the results, METTL16 was positively correlated with the expression of MKI67 and PCNA among most types of cancer. (Figure 4A). Furthermore, the expression of METTL16 is closely related to cell proliferation in BRCA and COAD. Next, we focused on the potential associations between METTL16 and the epithelial–mesenchymal transition (EMT) markers, N-cadherin (CDH2), Fibronectin 1 (FN1), Snail1 (SNAI1), Snail2 (SNAI2), twist family bHLH transcription factor 1(TWIST1) and Vimentin (VIM) [23]. The corresponding heat map showed that METTL16 was positively correlated with the expression of these EMT markers in COAD (Figure 4B). These results suggested that METTL16 may participate in significant biological differences in proliferation and metastasis.

### Enrichment analysis of METTL16 analysis

**Figure 5:**
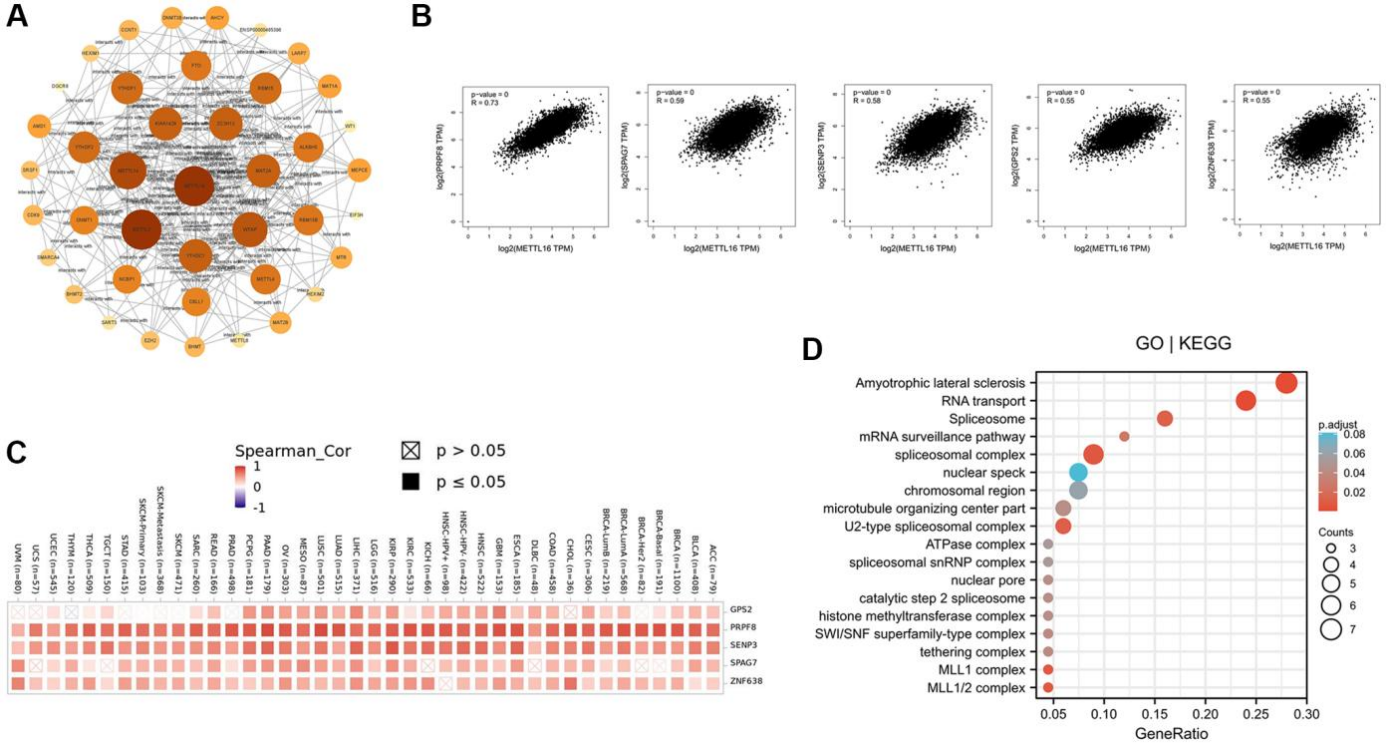
**Enrichment analysis of METTL16 -related gene.** (**A**) Protein–protein interaction network of METTL16 was performed via the STRING online resource. (**B**) Correlation of METTL16 and the top 5 genes (PRPF8, SPAG7, SENP3, GPS2 and ZNF638) in 33 types of cancer samples (**C**) The heat map of correlation between METTL16 and PRPF8, SPAG7, SENP3, GPS2 and ZNF638 in TCGA tumors. (**D**) GO|KEGG pathway analysis of the top 100 genes that associated with METTL16 expression.

In order to study the potential molecular mechanism of METTL16 gene in tumorigenesis and development, we constructed the protein–protein interaction (PPI) network and performed the METTL16 expression- correlated genes analysis. As shown in Figure 5A, we used STRING online tool to conclude a total of 40 METTL16 binding proteins. Subsequently, we used GEPIA2 tool to collect the expression data across all TCGA tumors and got the top 100 genes that most correlated with METTL16 expression. The GEPIA2 “Correlation Analysis” module showed that the top 5 genes are PRPF8(pre-mRNA processing factor 8) (R = 0.73), SPAG7(sperm associated antigen 7) (R = 0.59), SENP3(SUMO specific peptidase 3) (R = 0.58), GPS2(G protein pathway suppressor 2) (R = 0.55) and ZNF638(zinc finger protein 638) (R = 0.55) (Figure 5B). Next, we used TIMER2.0 tool to plot a heat map revealed that it existed strong positive correlations between METTL16 and the top 5 genes in most cancer types (Figure 5C). Exactly, previous studies have shown that PRPF8, SPAG7, SENP3 and GPS2 are associated with the occurrence of various cancers [24–28].

To further investigate the possible function of METTL16, we performed GO and KEGG pathway enrichment analysis of the top 100 correlated genes of METTL16 (Figure 5D). The enrichment of KEGG related to METTL16 is the amyotrophic lateral sclerosis signaling and RNA transport pathway. Moreover, the gene ontology enrichment analyses, such as biological process (BP), cellular component (CC) and molecular function (MF) related to METTL16 were mainly involved in spliceosome complex, chromosomal region. These results suggested the possible molecular mechanism of METTL16 in cancer pathogenesis.

### METTL16 promotes CRC cell proliferation and migration

**Figure 6:**
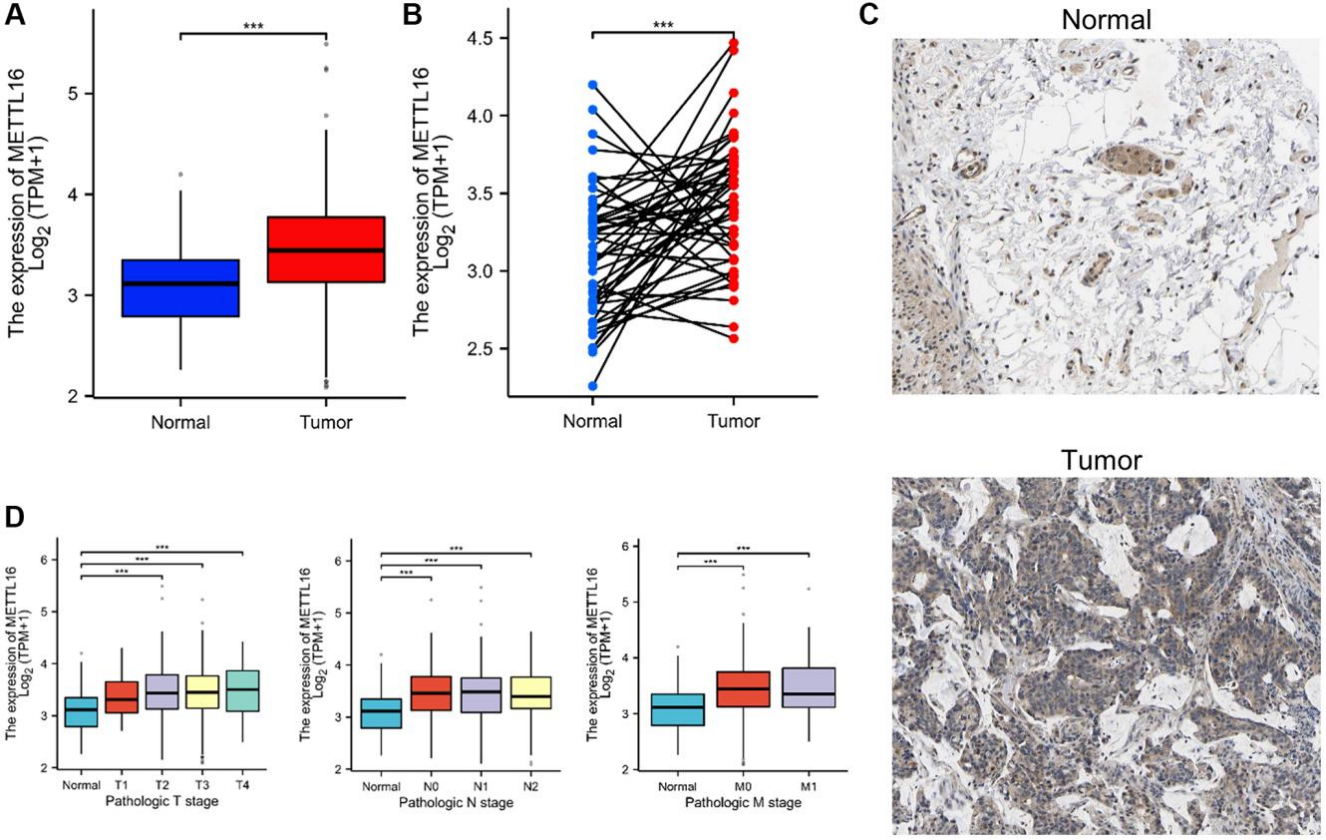
**METTL16 is upregulated in CRC.** (**A**, **B**) METTL16 expression in the TCGA CRC cohort. (**C**) Representative images of METTL16 IHC staining in CRC tissues and adjacent tissues. (**D**) Association of METTL16 mRNA expression with clinical stage grade.

Compared with normal tissues, TCGA databases showed that METTL16 was remarkably increased in CRC tissues (Figure 6A, 6B). The results of immunohistochemistry also verified the results of METTL16 high expression in CRC tissues (Figure 6C). As shown in Figure 6D, increased expression of METTL16 correlated significantly with the clinical stage grade. Differential analysis and prognostic analysis showed that METTL16 played an important biological role in CRC.

**Figure 7:**
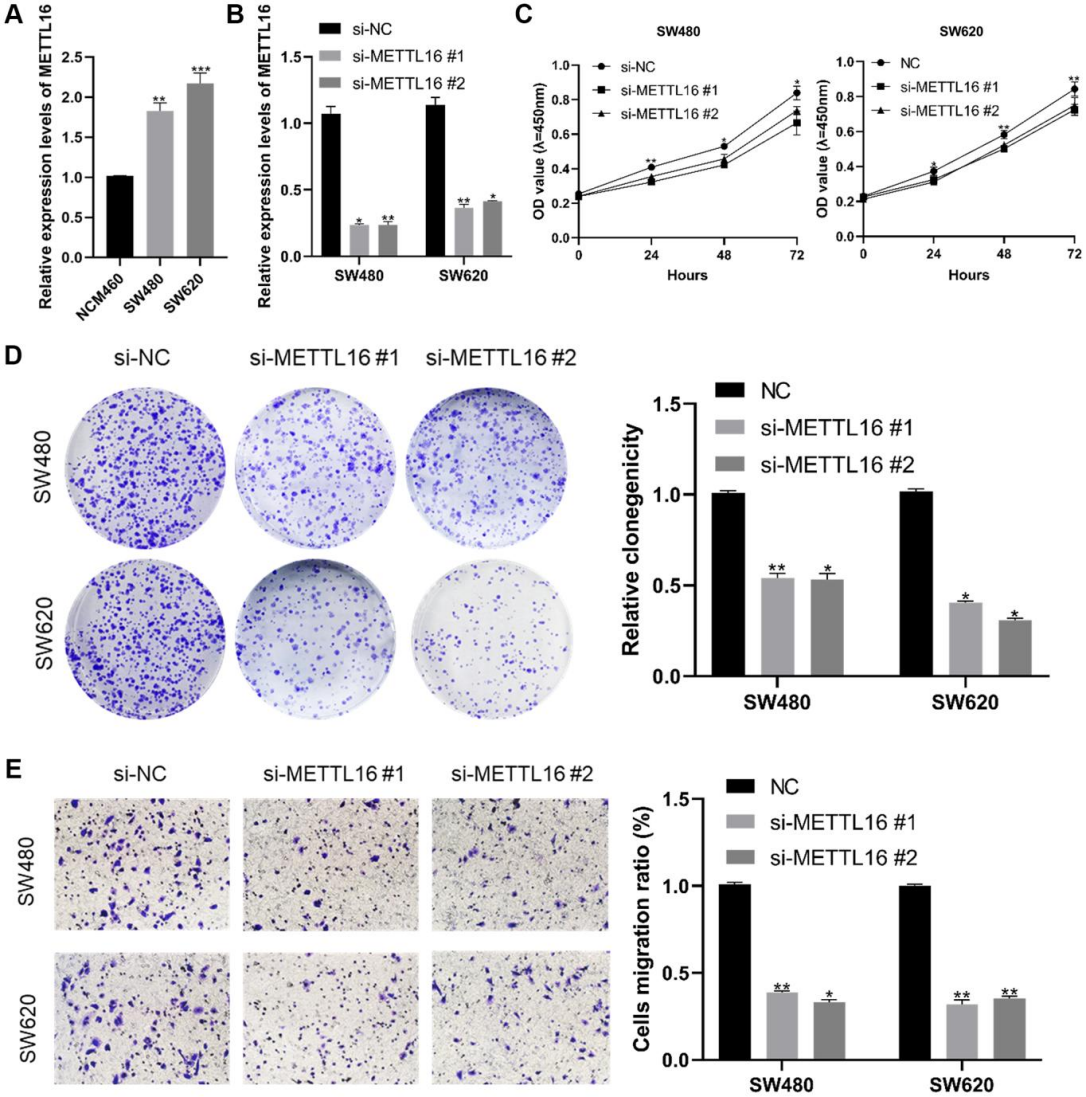
**Effect of METTL16 in CRC cell proliferation and migration.** (**A**) transcription level of METTL16 in CRC cell lines and NCM460. (**B**) transcription level of METTL16 in SW620 and SW480 were significantly downregulated by si-METTL16 transfection, respectively. (**C**, **D**) cell proliferation was assessed by CCK-8 and colony assays. (**E**) transwell assay employed to detect the migration ability of METTL16 knockdown cells. ^*^p < 0.05; ^**^p < 0.01; ^***^p < 0.001.

Our qRT-PCR validation of this finding in CRC cell lines (SW480 and SW620) and normal cells was consistent with the results from the database. The results show that METTL16 expression level was highly expressed in CRC cells compared with the normal cell line NCM460 (Figure 7A). Next, we confirmed the knockdown efficacy of si-METTL16 through qRT-PCR (Figure 7B). We performed CCK-8 and colony assays proving that eliminated METTL16 significantly decreased proliferation ability (Figure 7C, 7D). Results from transwell experiments showed that a decrease in the cell migration abilities in both SW480 and SW620 cell lines when METTL16 was knocked down (Figure 7E).

### CeRNA network construction in COAD

**Figure 8:**
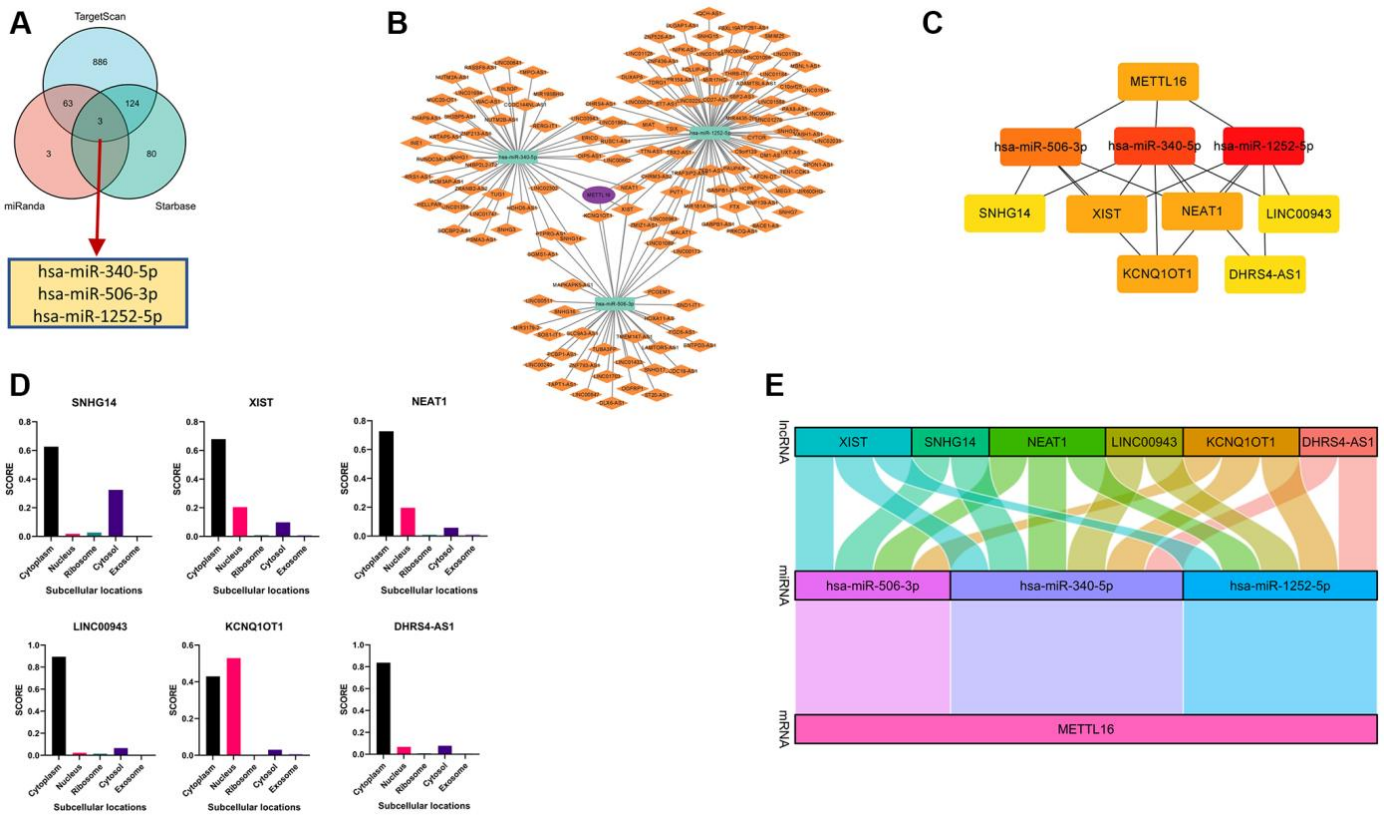
**Construction and correlation analysis of the ceRNA network.** (**A**) Venn graph showing the prediction results of METTL16 targets in Starbase, miRDB and TargetScan tools. (**B**) The triple regulatory network of ceRNA. The ellipses denote mRNA, rectangles denote miRNA and diamonds denote lncRNAs. (**C**) ten hub genes in this network. (**D**) The cellular localization for six hub-lncRNAs (XIST, SNHG14, NEAT1, LINC00943, KCNQ1OT1, DHRS4-AS1) was predicted using lncLocator. (**E**) The Sankey diagram of the lncRNA-miRNA-mRNA (METTL16) network in line with the ceRNA hypothesis.

Competing endogenous RNAs (ceRNAs) that can provide a framework to systematically interact with each other at post-transcription level by competitively binds shared miRNAs [29, 30]. There is growing evidence that ceRNA crosstalk can contribute to regulating their biological functions in carcinogenesis. So, we tried to analyze and construct a lncRNA-miRNA-mRNA ceRNA network involving METTL16. We verified miRNA-mRNA pairs from TargetScan, miRDB and R Starbase databases. By integrating the interactions among the above databases, we predict 1076, 72 and 208 METTL16 target miRNAs. To further identify competing miRNA-mRNA interactions, a combined study by Venn diagram to predict several common target miRNA genes in TargetScan, miRDB and Starbase software. We concluded the common miRNA predicted by three databases, including hsa-miR-340-5p, hsa-miR-506-3p and hsa-miR-1252-5p (Figure 8A). To establish the ceRNA triple regulatory network, we sought candidate lncRNAs that were only shared by the two databases (miRNet and Starbase) to enhance the veracity of the prediction. The results revealed that 45 target lncRNAs expression levels that are correlated with hsa-miR-340-5p, 37 target lncRNAs expression levels that are correlated with hsa-miR-506-3p, and 80 target lncRNAs expression levels that are correlated with hsa-miR-1252-5p (Figure 8B). The Cytoscape plug-in cytoHubba was used to determine the hub crucial regulatory network. The results showed that 6 lncRNAs (XIST, SNHG14, NEAT1, LINC00943, KCNQ1OT1, DHRS4-AS1), three miRNAs (hsa-miR-340-5p, hsa-miR-506-3p, hsa-miR-1252-5p) and METTL16 (Figure 8C). Moreover, considering that the underlying mechanisms were determined cellular localization of lncRNAs, we explored the subcellular localization of the above six lncRNAs by finding data in the lncLocator. The KCNQ1OT1 was mainly located in the nucleus and the other lncRNAs mainly located in cytoplasm (Figure 8D). Furthermore, we used GEPIA to find the connection relationship between lncRNAs expression and METTL16 expression. These data indicate that the above six lncRNAs can act as a ceRNA in the background of the ceRNA hypothesis. Finally, we construct ceRNA networks contain 15 pairs of lncRNA-miRNA-mRNA from the correlation analysis results (Figure 8E).

## DISCUSSION

Human Methyltransferase-like 16(METTL16) is an independent N6-methyladenosine (m6A) methyltransferase and it plays an important role in the process of cancers by forming m6A marks on S-adenosylmethionine (SAM) synthetase pre-mRNA and U6 small nuclear RNA (U6 snRNA). METTL16-dependent m6A modification activates or inhibits many biological processes including regulation of protein transport and ubiquitination, apoptosis, cell cycle, DNA-templated transcription, and actin cytoskeleton organization [31, 32]. The regulation of the METTL16 pattern participates in multiple biological processes and may leads to tumor initiation or progression when it affects tumor associated genes.

To assess the METTL16 across cancers more comprehensively, we decided to study the expression of METTL16 gene and its relationship with the prognosis of different cancer patients. Then we analyzed the expression and prognosis of the METTL16 gene in 33 tumors in TCGA database. Differential expression of METTL16 between tumor and normal tissues existed in many types of cancer. We found that compared with normal tissues, METTL16 gene expression showed high expression in some cancers, but low expression in the rest of the cancers from the database. We speculate that this is caused by the heterogeneity and complexity of different tumors. We also found that the poor OS was related to the overexpression level of the METTL16 gene in CRC and breast cancer. Some types of cancer have been reported that it exists a potential association between METTL16 and disease. In HCC, low METT16 expression was associated with the activation of multiple metabolic pathways and predicted poor OS [5]. In OV, the low expression of METTL16 also been determined among 17 m6A RNA methylation regulators [33]. However, the high expression of METTL16 has been linked with poor clinicopathological features and survival outcomes in breast cancer patients [34]. In consideration of the results that different tumors may have different degrees of the METTL16 involvement during the process of multiple cancer progression, we assumed that it could be an efficient strategy with clinical benefits to modulate METTL16 therapeutic expression according to different tumor types.

In addition, we investigated METTL16’s localization in the cells. It is essential for a complete understanding of functions of METTL16 that analysis of the localizations of proteins and their dynamics at the subcellular level. The distributed of METTL16 which was consistent with the results of a previous study [35]. The database showed that METTL16 mainly localized to the nucleoplasm. Subcellular localization is closely related to most biological processes such as binding to lncRNAs or miRNAs and the nuclear-cytoplasmic shuttling of transcription factors [36].

Genetic and epigenetic alterations have been found in every region of the protein virtually but only a handful of the mutation sites have been studied in depth in cancer progression. Mutations of R200 to glutamates abolish *in vitro* METTL16 methylation activity and reduce RNA binding. A similar effect has been observed for R203, R204 substitution [37]. Interestingly, the single mutation R200Q can interact with the RNA substrate and stabilize its conformation to increase m6A modification efficiency [38]. A number of challenges still remain to be overcome before the studies of genetic and epigenetic alterations can be applied into clinical practice. mutant-R200Q reactivation may potentially be used for treatment of human tumors via stabilization of m6A modification. Our studies also proved that METTL16 closely related with the immune infiltration in human cancers. However, further experimental researches are need to prove its function. In addition, we conducted *in vitro* experiments to verify the oncogenic role of METTL16 in CRC. Knockdown of METTL16 significantly suppressed CRC cancer cell growth and migration *in vitro*.

Competing endogenous RNAs (ceRNA) reveals a novel mechanism for RNA-RNA interactions. Long non-coding RNAs (lncRNAs) or circular RNAs (circRNAs) competitively binding shared miRNA to regulate downstream target genes [39]. To establish a ceRNA network of METTL16, we first used 3 databases jointly predicting upstream miRNAs (hsa-miR-340-5p, hsa-miR-506-3p and hsa-miR-1252-5p). Wan et al. [40] found that over expression of hsa-miR-340-5p can inhibits epithelial–mesenchymal transition in endometriosis. Lei et al. [41] reported that hsa-miR-506-3p can promotes the proliferation and metastasis of hepatocellular carcinoma by competing with EZH2. Then, we further predicted the upstream key lncRNAs (XIST, SNHG14, NEAT1, LINC00943, KCNQ1OT1, DHRS4-AS1). Interestingly, wo found that XIST, SNHG14, NEAT1, LINC00943, KCNQ1OT1 have pro-tumor functions in various cancers but DHRS4-AS1 can inhibit tumor development [42–47]. In addition, some studies concluded that the long noncoding RNAs MALAT1and XIST were identified METTL16-bound RNAs [48, 49]. XIST can directly bound with METTL16 playing a variety of biological roles [2]. While this research further supports the feasibility of our network model, further biological experiments are required to confirm our prediction. We also concentrate on protein-protein interaction network, co-expression genes and the relationships between CNV were all investigated in depth. These results confirm the reliability of the pan-cancer bioinformatics analysis.

However, there were still some limitations to this study even though we integrated information from different databases. For example, despite the fact that METTL16 expression was strongly related to immunity and clinical survival, the potential mechanisms of immune regulation need to be further studied. In addition, while the bioinformatic analysis has provided important insight into our initial understanding of the role of METTL16, *in vivo* and vitro experiments are still needed to prove our results on the function of METTL16 and explore more effective treatments. Overall, we conducted original research that concluded the values of METTL16 in various cancers. Our findings revealed the critical roles of METTL16 in tumorigenesis, prognosis, location and immunology. We confirmed that our work may provide a comprehensive understanding of METTL16 to guide treatment approaches.

## Supplementary Materials

Supplementary Figures
