# Supplementary material for: Pan-cancer analysis of Methyltransferase-like 16 (METTL16) and validated in colorectal cancer
Source: Aging (Albany NY). 2025 Feb 27;17(2):588–606. doi: 10.18632/aging.206210 (PMC11892929; doi:10.18632/aging.206210)
Supplement: Supplementary Figures [file aging-17-206210-s001.pdf]

SUPPLEMENTARY FIGURES

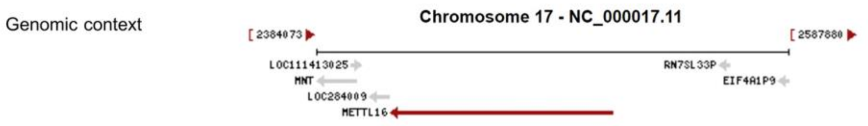

Annotation release	Status	Assembly	Chr	Location
110	current	GRCh38.p14 (GCF_000001405.40)	17	NC_000017.11 (2415715..2511888, complement)
110	current	T2T-CHM13v2.0 (GCF_009914755.1)	17	NC_060941.1 (2304729..2400696, complement)
105.20201022	previous assembly	GRCh37.p13 (GCF_000001405.25)	17	NC_000017.10 (2319343..2415182, complement)

Supplementary Figure 1. Structural characteristics of METTL16.

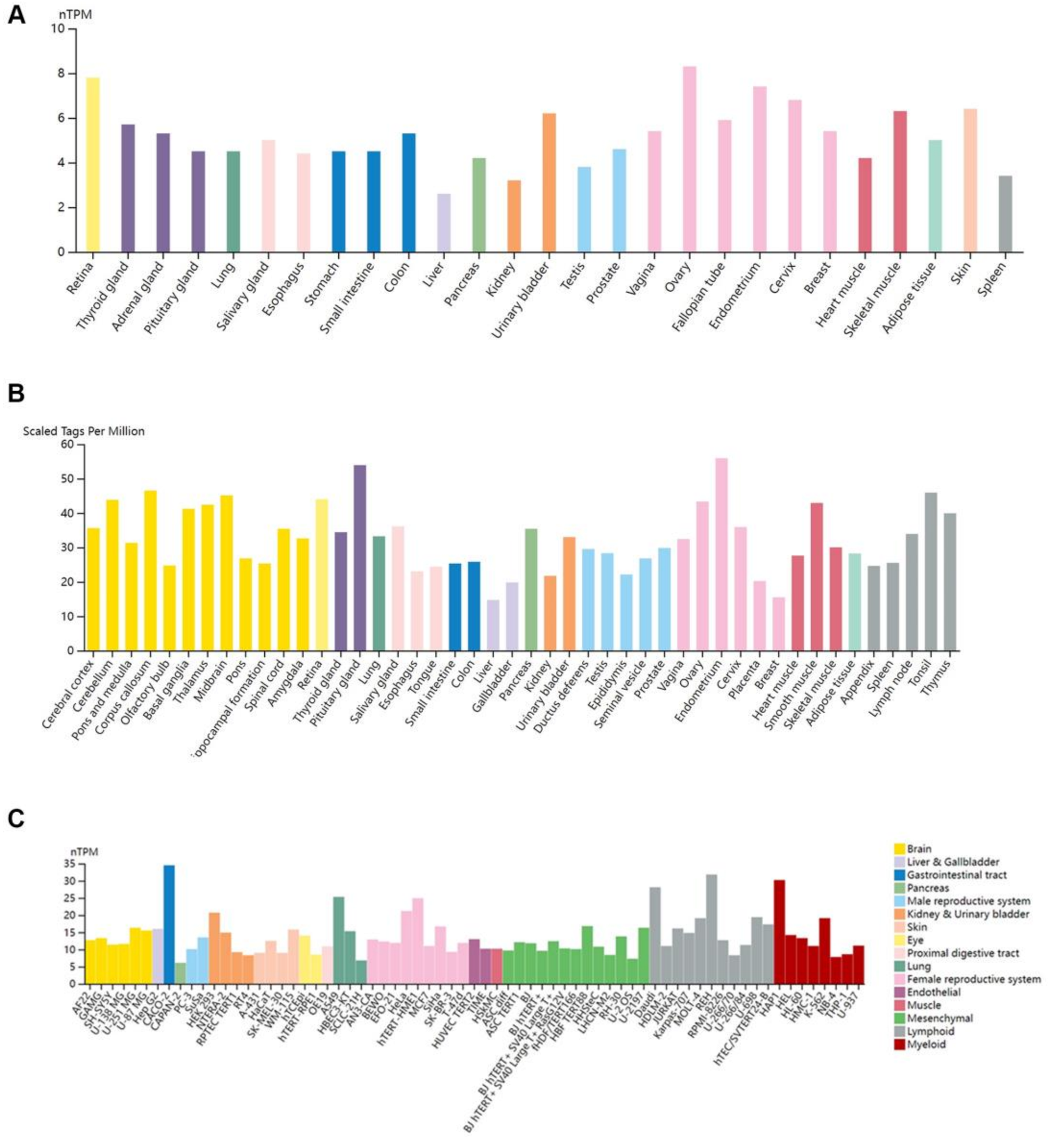

Supplementary Figure 2. Expression level of METTL16 in different cells and tissues. (**A**) HPA tissue dataset; (**B**) Tissue consensus dataset; (**C**) Cell line dataset.

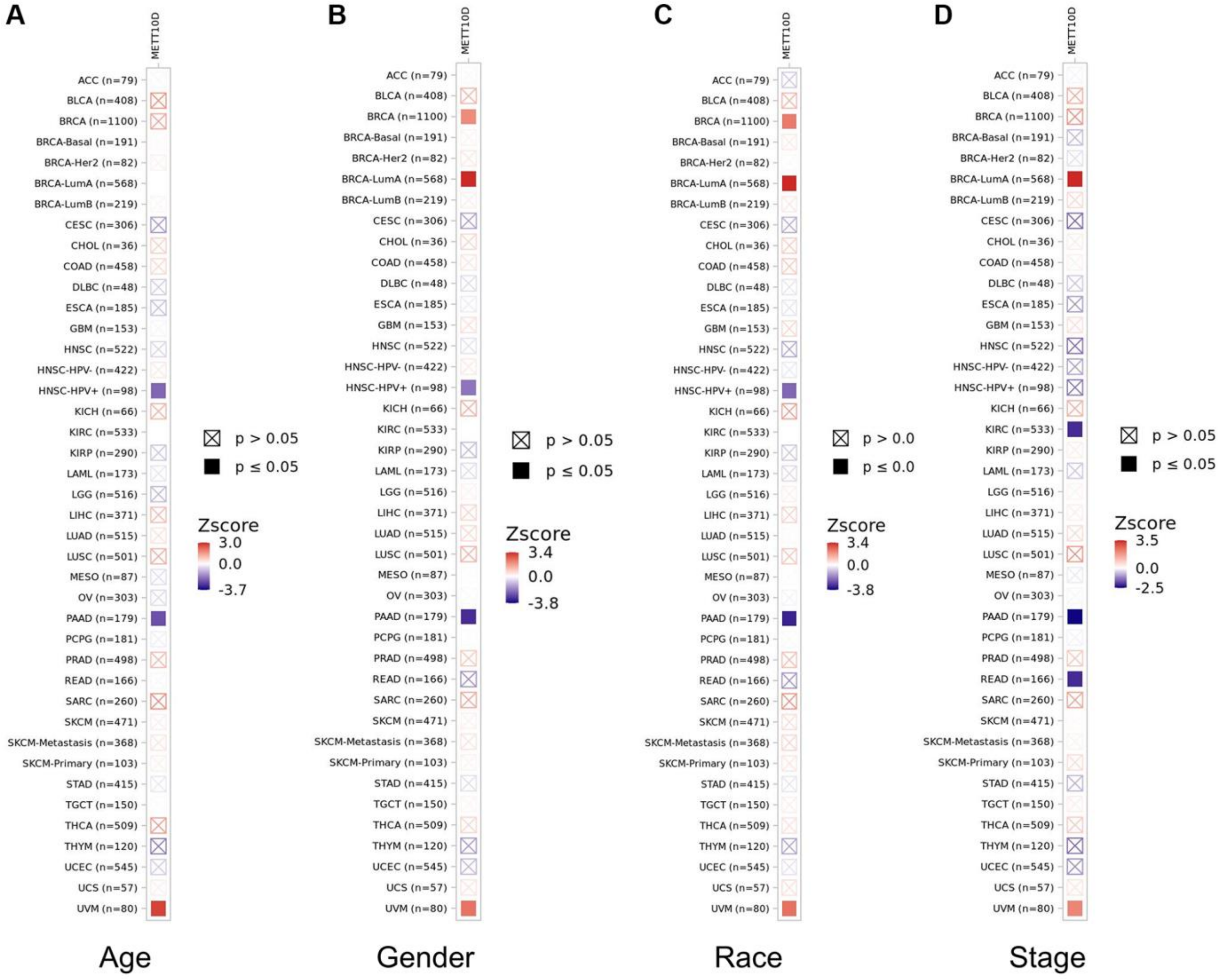

Supplementary Figure 3. The relationship between METTL16 gene expression with age (**A**), gender (**B**), race (**C**), and stage (**D**).

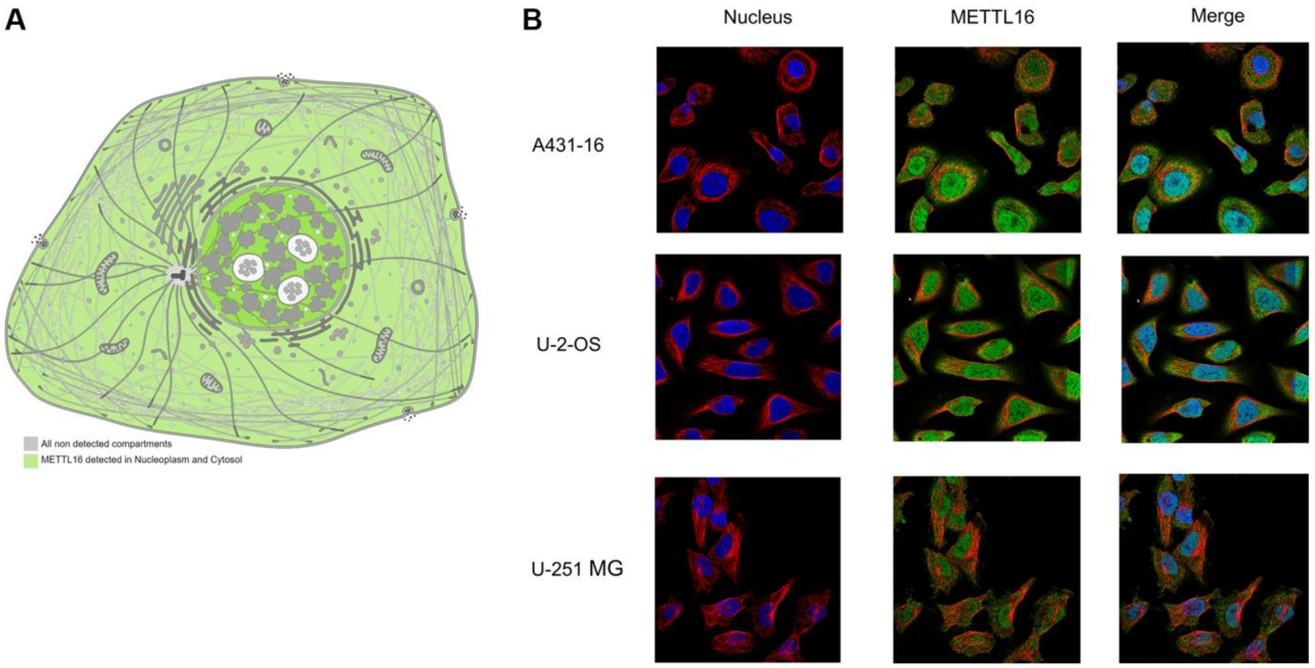

Supplementary Figure 4. The subcellular distribution of METTL16 (**A**) and the nucleus are marked in blue, the METTL16 marked in green, the microtubules are marked in red (**B**).

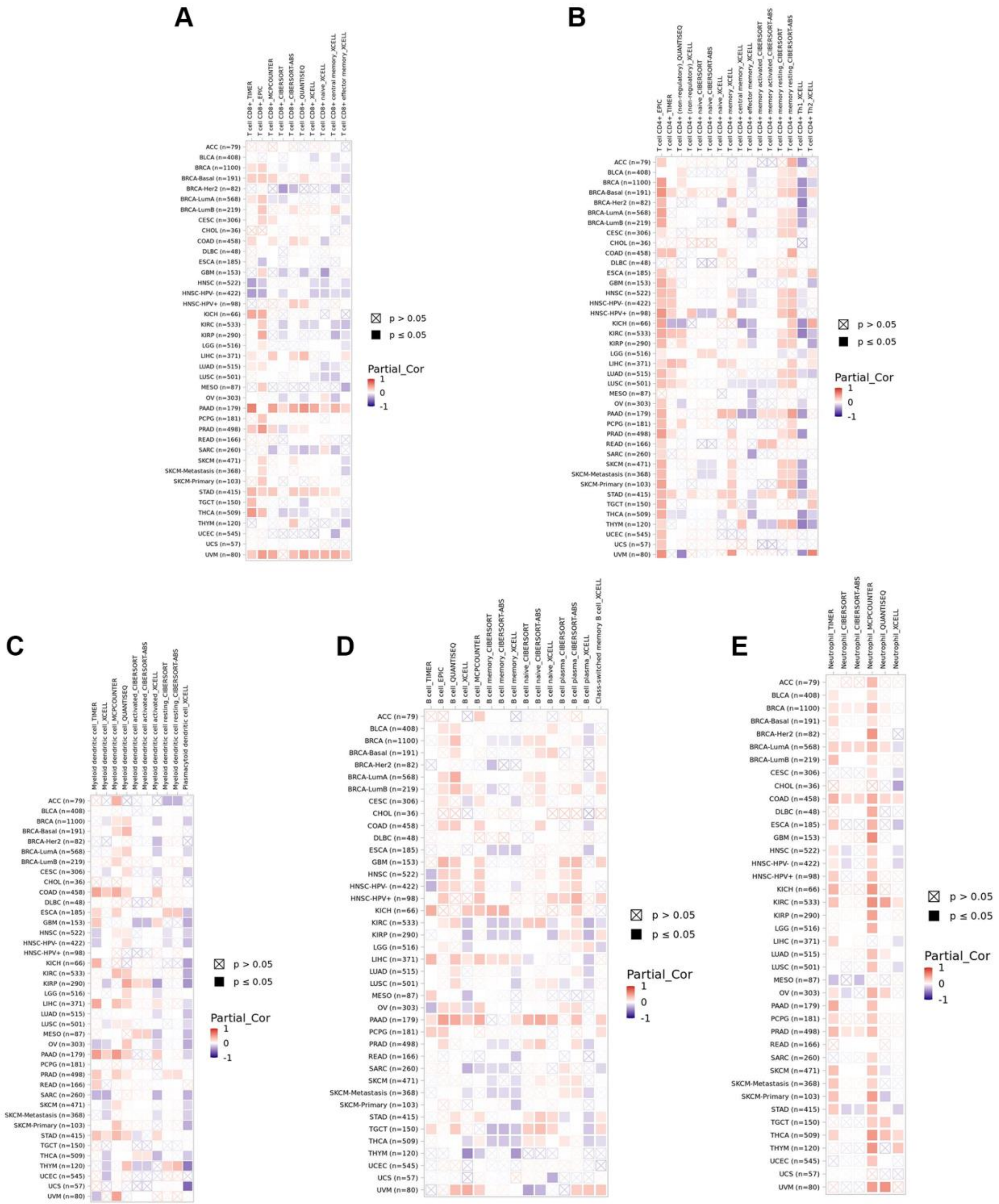

Supplementary Figure 5. Correlation analysis between METTL16 expression levels and the expression degree of immune cells, including CD8+ T cell (**A**), CD4+ T cell (**B**), Myeloid (**C**), B cell (**D**), Neutrophil (**E**).
